# Pomegranate–Quinoa-Based Agroforestry System: An Innovative Strategy to Alleviate Salinity Effects and Enhance Land Use Efficiency in Salt-Affected Semiarid Regions

**DOI:** 10.3390/plants13182543

**Published:** 2024-09-10

**Authors:** Ilham Abidi, Khalid Daoui, Aziz Abouabdillah, Didier Bazile, Abdel Aziz Hassane Sidikou, Loubna Belqadi, Hamid Mahyou, Si Bennasseur Alaoui

**Affiliations:** 1 Hassan II Institute of Agronomy and Veterinary Medicine, Rabat 10112, Morocco; aa.sidikou@gmail.com (A.A.H.S.); l.belqadi@gmail.com (L.B.); b.alaoui@iav.ac.ma (S.B.A.); 2 National Institute for Agricultural Research, Regional Agricultural Research Center, Meknès 50000, Morocco; daoui_khalid@yahoo.fr; 3 Ecole Nationale d’Agriculture de Meknès, Meknès 50001, Morocco; aziz.abouabdillah@gmail.com; 4 CIRAD, UMR SENS, F-34398 Montpellier, France; didier.bazile@cirad.fr; 5 UMR SENS, CIRAD, IRD, University Paul Valery Montpellier 3, University Montpellier, F-34090 Montpellier, France; 6 National Institute for Agricultural Research, Regional Agricultural Research Center, Oujda 60000, Morocco; hamid.mahyou@inra.ma

**Keywords:** *Chenopodium quinoa* Willd., grain yield, intercropping system, land equivalent ratio, pomegranate trees, salt stress

## Abstract

Salinity is a major problem, impeding soil productivity, agricultural sustainability, and food security, particularly in dry regions. This study integrates quinoa, a facultative halophyte, into a pomegranate-based agroforestry with saline irrigation in northeast Morocco. We aim to explore this agroforestry model’s potential in mitigating salinity’s effects on quinoa’s agronomic and biochemical traits and evaluate the land equivalent ratio (LER). Field experiments in 2020 and 2021 used a randomized block design with three replicates, including monocropping and agroforestry systems, two salinity levels (1.12 and 10.5 dS m^−1^), four quinoa genotypes (Titicaca, Puno, ICBA-Q4, ICBA-Q5), and a pomegranate control. Salinity significantly decreased total dry matter (40.5%), root dry matter (50.7%), leaf dry matter (39.2%), and root-to-shoot ratio (7.7%). The impact was more severe in monoculture than in agroforestry, reducing dry matter (47.6% vs. 30.7%), grain yield (46.3% vs. 26.1%), water productivity (47.5% vs. 23.9%), and total sugar (19.2% vs. 5.6%). LER averaged 1.86 to 2.21, indicating 86–121% higher productivity in agroforestry. LER averaged 1.85 at 1.12 dS m^−1^ and 2.18 at 10.5 dS m^−1^, reaching 2.21 with pomegranate-ICBA-Q5 combination. Quinoa–pomegranate agroforestry emerges as an innovative strategy, leveraging quinoa’s salt resistance and agroforestry’s potential to mitigate salinity impacts while enhancing land use efficiency.

## 1. Introduction

Salt-affected landscapes cover 1257 million hectares worldwide, representing about 8.5% of the land area across 118 countries with vast areas remain uncultivated in arid and semiarid regions [1,2]. In irrigated areas, salt-induced land degradation leads to an annual productivity loss of approximately USD 27.3 billion [3]. In addition to this, climate crisis is continuously amplifying soil and water salinization, impeding soil productivity, agricultural sustainability, and food security, particularly in arid and semiarid regions [4,5]. Furthermore, intensive monoculture exacerbates soil and water salinization. It covers 80% of the world’s arable land [6], making it susceptible to climate change and yield losses from pests and diseases. Sole cropping systems also degrade soil through improper agrochemical uses, heavy mechanization, and unbalanced crop rotations focused on cash crops. In response to salt pressure, plants endure a multitude of morphological, physiological, and metabolic changes, leading to substantial reductions in crop yield and quality [7,8].

Halophytes species adapt to salinity by adjusting internal water relations, sequestering ions in vacuoles, accumulating compatible organic solutes, developing succulence, and using salt-secreting glands and bladders [7,8,9,10]. Many halophytes, including quinoa, demonstrate high K+/Na+ selectivity to sustain cellular functions and growth under saline conditions [11,12]. This adaptation involves a combination of efficient ion transport mechanisms—primarily sodium (Na+) exclusion and selective potassium (K+) uptake—along with maintaining favorable K+/Na+ ratios in the cytoplasm through cytoplasmic homeostasis and sodium compartmentalization in vacuoles [13]. These strategies allow quinoa to thrive in saline environments by minimizing the detrimental effects of Na+ while ensuring sufficient K+ for essential cellular processes. Additionally, quinoa reduces transpiration rates by closing its stomata to conserve water [14]. This response decreases leaf gas exchange, stomatal conductance, and overall transpiration under saline conditions [15], ultimately improving photosynthetic water use efficiency [16,17]. Furthermore, under saline conditions, halophytes experience elevated ROS production that they manage effectively, using these molecules as signaling agents to activate stress adaptation pathways [18]. For instance, ROS can trigger the activation of antioxidant defenses and the expression of stress-responsive genes. These antioxidants neutralize excess ROS, protecting cells from oxidative damage. The key lies in maintaining a balance between ROS production and scavenging, enabling halophytes to leverage ROS for signaling while minimizing their noxious effects [19].

This ability makes halophytes advantageous for bioremediating salt-affected areas [20,21]. On the other hand, in dry and salt-prone regions, managing saline water can alleviate freshwater scarcity and enhance overall water productivity to meet the food demands of a rapidly growing population [22,23]. Furthermore, implementing salt-resilient systems that balance productivity and sustainability is crucial [24,25].

Over the past four decades, managing salt-affected lands has increasingly focused on strategies such as tree-based cropping, saline pastures, and biosaline agriculture [19,20]. Agroforestry in saline environments enhances land-use efficiency and supports vulnerable populations [26]. The soil–plant–atmosphere continuum (SPAC) illustrates the interconnected flow of water from soil through plants to the atmosphere, shaped by the interactions between trees, crops, and the environment. Trees access deep soil water and redistribute it to nearby crops, especially during dry periods [27]. Additionally, trees improve nutrient cycling, soil structure, and contribute organic matter, leading to more efficient water use and better plant growth [28]. Furthermore, positive interactions like moderate shading create a more stable microclimate for understory plants, by conserving soil humidity levels and reducing temperature fluctuations, which can lead to better growth conditions [29,30,31]. Furthermore, intercropping plants with legumes enhances soil nitrogen availability, benefiting neighboring plants and contributing to improved soil fertility through symbiotic relationships with nitrogen-fixing bacteria [32,33,34]. Additionally, these interactions can support beneficial soil microorganisms, such as plant-growth-promoting rhizobacteria (PGPR), enhancing nutrient uptake, disease resistance, and overall plant health [34].

Biosaline agroforestry combines the principles of agroforestry with the integration of salt-resilient species [35,36], addressing the needs of climate change mitigation and adaptation on salt-affected soils [37]. It can improve soil health [38], boost biodiversity, generate economic returns, and promote sustainable agricultural practices in saline environments [39]. This approach offers opportunities for farmers to integrate salt-resistant crops into agroforestry systems under saline irrigation when fresh water is scarce and conventional farming is unproductive [22,40].

The net productivity of halophyte-based agroforestry hinges on the dynamic interactions among its components across time and space, particularly in their utilization of shared resources like water, nutrients, and light. Species may compete when their demands for these limited resources overlap temporally and spatially [41]. However, positive interactions such as facilitation or complementarity can also occur if they utilize the available resources in separate ways [24,25]. In this context, Bayala et al. (2020) [42] asserted that intercropped species can enhance the acquisition of limited natural resources through complementary root distributions and water-sharing mechanisms. Indeed, while trees may compete with crops for soil resources, they can also provide shade, improving crop growing conditions [43], by reducing evapotranspiration and buffering temperature fluctuations [30,44]. However, shade differently affects crop yield depending on the shading’s intensity [31]. Shade reduces maize grain yield more significantly than soybean, whereas intercropping species under saline irrigation fosters positive synergies, achieving a land equivalent ratio of up to 1 [34,35,36,37,38].

In our study, we envision combining the positive effects of agroforestry with the high salt resistance of halophytic crops by integrating quinoa into pomegranate-based agroforestry. Indeed, quinoa has a unique versatility across diverse climates by exhibiting resilience to various abiotic stresses, including drought, heat, frost, and salinity [22,45,46,47,48]. Its unique versatility results in its efficient system adjusting osmotically and reducing its transpiration to maintain a positive water balance in response to salinity [49]. Its response to salinity stress is strongly genotype dependent [50,51]. Quinoa is considered a novel and healthy food thanks to its exceptional nutritional profile [52]. Quinoa seeds are gluten-free; rich in vitamins, high-quality fat, dietary fiber, and prominent protein (9–23%); and have a balanced amino acid profile [53,54,55,56,57]. Moreover, quinoa contains numerous bioactive compounds that confer medicinal properties, including phytosterols, saponins, phenolic compounds, phytoecdysteroids, polysaccharides, and betalains [58,59]. Compared to cereal grains such as barley, rice, maize, and oats, quinoa excels with a higher total protein content [60]. The resilience and high nutritional quality of quinoa have positioned it as a potential strategic crop for food and nutritional security [61]. It is recognized as “one of the grains of the 21st century”, poised to meet the needs of an expanding global population [62].

This study aims to explore the potential of halophytes-based agroforestry as an innovative option for enhancing salt-affected land use efficiency under saline conditions. We integrate quinoa, as a facultative halophyte, into a pomegranate-based agroforestry system under supplemental saline irrigation in the northeastern region of Morocco. Our objectives include assessing the overall performance of this new system while determining the land equivalent ratio. We will also investigate how intercropping quinoa with pomegranate trees mitigates the impact of salinity on the agronomic and biochemical traits of different quinoa varieties, seeking the optimal combination for local farmers dealing with salinity challenges. To accomplish this, we will compare the variations in these parameters between saline and control treatments within each cropping system. We hypothesize that (1) Intercropping quinoa with pomegranate trees under saline irrigation can serve as an efficient agroforestry model, enhancing the land use efficiency and the productivity of the whole system; (2) Due to the positive relationships of facilitation and complementarity between quinoa and pomegranate trees, quinoa–pomegranate agroforestry can mitigate the negative effects of salinity on both pomegranate and quinoa productivity compared to growing them separately. On the other hand, (3) the response to salinity will also depend on climatic conditions and the specific combinations of quinoa varieties and pomegranate trees. We aim to (i) assess the effectiveness of the quinoa–pomegranate agroforestry system and determine the optimal pomegranate tree–quinoa cultivar combination using the land equivalent ratio (LER), and (ii) compare the quinoa agronomic and biochemical traits variation under saline irrigation in both agroforestry and sole cropping systems.

## 2. Results

### 2.1. Quinoa Growth Parameters

Table 1 summarizes the results of the ANOVA (analysis of variance) for all investigated parameters as affected by irrigation water salinity, cropping systems, quinoa genotypes, and their interactions. Significant variations were observed in RDM (−11.8%), LDM (194%), SLA (−66.57%), and root/shoot ratio (−15.4%) within AFS compared to SCS. Variations according to salinity levels were −10% in height, −40.5% in TDM, −50.7% in RDM, −39.2% in LDM, and −7.7% in root/shoot ratio within EC2 (10.5 dS m^−1^) compared to EC1 (1.12 dS m^−1^). Furthermore, Puno averaged the high values in height (131.7 cm), TDM (80.3 g plant^−1^), and RDM (8.5 g plant^−1^), while low averages in all parameters, except root/shoot, were recorded by ICBA-Q5.

### 2.2. Quinoa Grain Yield and Yield-Related Components

The results revealed highly significant impacts of irrigation water salinity and quinoa genotypes on all investigated agronomic traits. Total dry matter (TDM), grain yield, and quinoa water productivity (QWP) did not vary significantly between the two cropping systems, whereas the experimental year had a significant effect on all parameters, except the harvest index (HI). Regarding interactions, (CS × EC), (EC × Variety), and (CS × EC × Variety) significantly affected nearly all parameters (Table 2). Table 3 synthetizes the variation in all agronomic parameters under saline irrigation water (10.5 dS m^−1^) compared to the control (1.12 dS m^−1^), reported in Table S1. We observed that high salinity level reduced all parameters, except the harvest index within the two cropping systems and across both experimental years. The losses were more pronounced within sole cropping system (SCS) than agroforestry (AFS). In 2020, the reductions due to salinity in dry matter, grain yield, thousand-kernel weight, and quinoa water productivity were (30.2% vs. 45.6%), (33.3% vs. 46.7%), (10.3% vs. 14.8%), and (23.9% vs. 44.4%), respectively, in AFS compared to SCS. In 2021, the related losses were (31.3% vs. 47.1%), (24.2 vs. 45.9%), (7.7% vs. 16%), and (23.9% vs. 46.4%). Conversely, high irrigation water salinity increased the harvest index by 11.5% when quinoa varieties were intercropped with pomegranate trees. Furthermore, the impact of salinity on all parameters was quinoa genotype dependent. Titicaca was the most affected variety by salinity, particularly during the first experimental season, whereas ICBA-Q5 exhibited the lowest percentage of loss in most parameters (Table 2).

### 2.3. Pomegranate Yield and Land Equivalent Ratio (LER)

**Figure 1:**
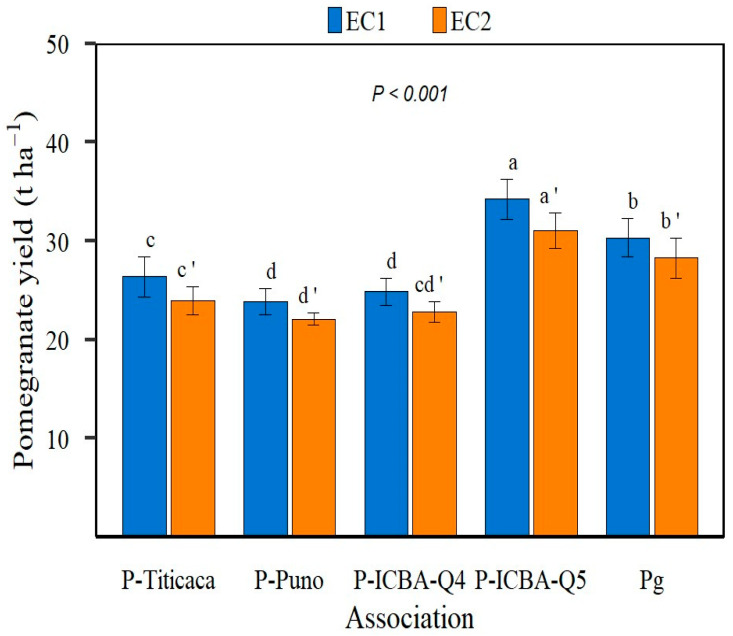
Pomegranate yield (t ha^−1^) as affected by water salinity level (*p* < 0.001) and by pomegranate–quinoa associations (P-Titicaca, P-Puno, P-ICBA-Q4, P-ICBA-Q5) compared to pomegranate orchard (Pg). Vertical bars denote standard deviations (n = 6). For electrical conductivity of control treatment (EC1 = 1.12 dS m^−1^), means followed by the same lowercase letter are not significantly different. Similarly, for saline water treatment (10.5 dS m^−1^), means followed by the same lowercase letter (with apostrophe) are not significantly different.

The average pomegranate yields were significantly higher within EC1 than EC2 (27.92 t ha^−1^ vs. 25.62 t ha^−1^, *p* < 0.001). Furthermore, significant interactions between water salinity levels and cropping systems were noted (*p* < 0.001). Pomegranate yields decreased differently under saline irrigation (EC2), depending on pomegranate–quinoa associations or pomegranate orchard. For instance, when associated with ICBA-Q5, pomegranate yield decreased by 10% under EC2. Furthermore, within the same water salinity level, significant differences in pomegranate yield among different pomegranate–quinoa associations were recorded (*p* < 0.001). In fact, it ranged from 23.9 t ha^−1^ (*p*-Puno) to 34.2 t ha^−1^ (*p*-ICBA-Q5) with EC1 and from 22.1 t ha^−1^ (P-Puno) to 31 t ha^−1^ (P-ICBA-Q5) with EC2. Significant variations in pomegranate yield were recorded among quinoa varieties–pomegranate associations, and pomegranate orchard (*p* < 0.001). Under both water regimes, the P-ICBA-Q5 combination resulted in an increase in the average pomegranate yield by 13% and 10% compared to the pomegranate orchard under EC1 and EC2 conditions, respectively (Figure 1).

The contribution of quinoa varieties and pomegranate trees to the overall productivity of the agroforestry system were evaluated by calculating the partial land equivalent ratios of each component: land equivalent ratio for quinoa varieties (LER_Quinoa_) and for pomegranate trees (LER_Pomegranate_). LER_Quinoa_ and LER of the whole system were significantly affected by irrigation water salinity (*p* < 0.001) and were 36.2% and 20% higher under saline conditions (10.5 dS m^−1^) compared to the control (EC1 = 1.12 dS m^−1^), respectively. Furthermore, LER_Pomegranate_ and LER varied significantly depending on the quinoa variety associated with pomegranate trees. The higher averages were recorded by P-ICBAQ5 association for both parameters and were 1.11 and 2.21, respectively. LER_Quinoa_ and LER were significantly influenced by the interactions between salinity and quinoa variety intercropped with pomegranate trees (*p* < 0.001), whereas LER_Pomegranate_ varied significantly with the interaction between experimental season and irrigation water salinity. LER_Quinoa_ and LER_Pomegranate_ averaged from 1.08 to 1.15 and from 0.79 to 1.11, respectively. LER of the whole system was always higher than 1 and averaged from 1.86 to 2.21 (Table 4).

### 2.4. Nutritional, Mineral, and Saponin Content in Quinoa Seeds

The results depict substantial variations in mineral (F = 178.2), protein (F = 84.5), total sugar (F = 39.9), and saponin (F = 9.6) accumulation across the cropping systems. Total sugar was 13% lower under saline irrigation compared to the control. Quinoa genotype significantly influenced all seed nutritional components (*p* < 0.01), while the experimental year affected only mineral content (Table 5). Significant differences in seed mineral and protein content were found based on the cropping system and the experimental seasons interaction (Year × CS, *p* < 0.001), and in seed mineral content under the interaction between salinity levels and the experimental season (Year × EC, *p* < 0.001). Total sugar and saponin content varied significantly with irrigation water salinity and cropping systems (CS × EC, *p* < 0.001). Additionally, mineral content, fat content, and crude cellulose (CB) showed notable variation due to the interactions between quinoa genotypes and irrigation water salinity (EC × Variety, *p* < 0.01) (Table 5).

Seed mineral content was 5.6% lower in 2021 for both AFS and SCS (Figure S1a). Protein content, fat content, and crude cellulose content did not show any significant differences with irrigation water salinity for both cropping systems across the two experimental seasons (Figure S1b–d). However, a significant increase in protein content was recorded in AFS compared to SCS (17.1% vs. 15.7%) (Figure S1b). Additionally, in SCS over the two experimental years, the average total sugar significantly decreased by 20% under salinity compared to the control (Figure S1e). Seeds saponin content is quinoa genotype dependent, averaging from 0.4% (ICBA-Q5) to 0.6% (Titicaca) (Table S2). Nonetheless, significant differences were noted between the two cropping systems (*p* < 0.05) in 2020, but not in 2021 (*p* = 0.702). Similarly, seeds saponin content was significantly affected by salinity in AFS, unlike in the SCS system, over both seasons (Figure S1f).

Unlike salinity, all other factors significantly affected phosphorus content in quinoa seeds, with the cropping system contributing most to this variation (F = 34.8, *p* < 0.001). In addition, the interactions CS × EC, CS × Variety, EC × Variety, and CS × EC × Variety significantly influenced this parameter (Table 6). Remarkably, over both experimental seasons, seeds phosphorus content increased under saline irrigation in AFS, contrary to SCS (Table S2). Seed potassium content significantly decreased by salinity (*p* < 0.05) and variety (*p* < 0.001) across both cropping systems. Substantial differences in seeds sodium content between AFS and SCS (33.2 vs. 27.2, *p* < 0.01) were observed (Table 6). Notably, this parameter was 20% higher under saline irrigation in SCS over 2021 (Table S2). Under saline irrigation, the ratio K/Na was 6.5% and 17.4% higher compared to the control in AFS over 2020 and 2021, respectively (Table S2). Titicaca averaged a low K/Na ratio (24) while Puno, ICBA-Q4, and ICBA-Q5 did not exhibit any remarkable differences (Table 6).

### 2.5. Correlation Matrix and Principal Component Analysis

**Figure 2:**
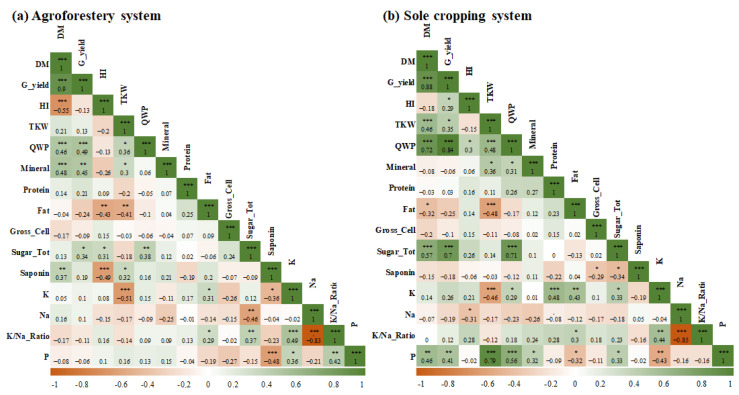
Person’s correlation matrix for the monitored parameters in agroforestry (**a**) and sole crop systems (**b**). Values in the matrix represent Pearson’s correlation coefficient. *, **, and *** indicate the significance of the correlation coefficient at *p* < 0.05, 0.01, and 0.001, respectively. The matrix graphical representation was carried out using the corrplot package of R programming language.

The correlation matrix exhibited more significant correlations among the evaluated quinoa parameters in SCS than in AFS (Figure 2). Indeed, quinoa dry matter, grain yield, water productivity, and seeds contents of fat and total sugar were significantly correlated with more parameters in SCS than in AFS. Under agroforestry conditions, the grain yield was correlated with the dry matter (r = 0.90), crop water productivity (r = 0.49), mineral content (r = 0.45), and grains sugar content (r = 0.34). On the other hand, under monocropping conditions, the grain yield was significantly correlated with the dry matter (r = 0.88), harvest index (r = 0.29), thousand-kernel weight (r = 0.35), crop water productivity (r = 0.84), seeds contents of total sugar (r = 0.70), and phosphorus (r = 0.41). Furthermore, quinoa water productivity was only significantly correlated with the grain yield and the grains sugar content (r = 0.38) in AFS, whereas, under SCS conditions, it was significantly correlated to grain yield, mineral content (r = 0.31), total sugar (r = 0.71), potassium (r = 0.29), and phosphorus (r = 0.56).

**Figure 3:**
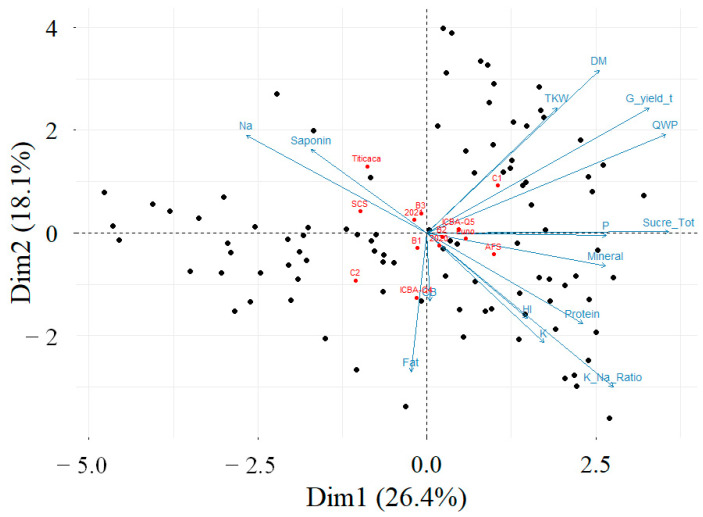
PCA–biplot projection of individuals (black points), factors (red points), and variables (blue arrows) on the main plan for the investigated parameters. The plot was designed with the “Factoextra” package of R programming language.

The first plane of the PCA explained 44.5% of the data variability (Figure 3). Such a variability is significant since it is greater than the reference value of 23.7% (Husson, Lê, and Pagès 2017). Grain yield, quinoa water productivity, and seeds content of total sugar mostly contributed to the construction of the first axis of the PCA. The cumulative contribution of those three variables for the construction of the first PCA dimension amounted to 40.8%. Among all the evaluated parameters, only the seeds content in saponin, sodium, fat, and gross cellulose were negatively correlated to the first dimension of the PCA. However, the correlations between the seeds contents in fat and gross cellulose with the first dimension were not significant. Concerning the second component, it was mainly explained by the grain yield, thousand-kernel weight, seeds total sugar content, seeds potassium content, seeds mineral matter content, and the quinoa water productivity. Grain yield, thousand-kernel weight, and quinoa water productivity were positively correlated with the second dimension of the PCA. However, the seeds content in potassium and mineral matter were negatively correlated with the second dimension of the PCA. Furthermore, the first plane of the PCA contrasted with the cropping systems and the salinity levels. Indeed, quinoa plants grown under the lowest salinity level (C1) resulted in the highest levels of grain yield, dry matter, thousand-kernel weight, and quinoa water productivity. In contrast, the plants grown under C2 conditions recorded the highest level of seed fat content.

## 3. Discussion

### 3.1. Quinoa Growth Parameters at Full Flowering Stage

Our findings clearly show that saline irrigation significantly influenced all the investigated growth parameters of quinoa (Table 1). This result is in line with the authors of [63,64], who found that increasing salinity irrigation water decreased the morphological and physiological traits. Furthermore, the reduced root development in AFS (−11.8%) suggests that quinoa does not compete with pomegranate for nutrients and water. In contrast, when grown alone, quinoa developed its roots more extensively, likely to better compete for the limited water and nutrients caused by salinity. The slight increase in TDM with AFS contrasts with Ben Zineb et al. (2022) [65], who specified that dry matter production at the flowering stage of barley, durum wheat, and chickpea was significantly lower in AFS than SCS. Meanwhile, LDM increased by close to three times in AFS, whereas SLA was 66.6% lower in AFS, maintaining an LA significantly equivalent between AFS and SCS. In our study, quinoa developed thicker and tougher leaves to adapt to shade caused by pomegranate trees. This finding is well confirmed by a lower root/shoot ratio in AFS compared to SCS. Root to shoot describes the relative biomass allocation between the roots and the aboveground parts (shoots). Plants with a low root-to-shoot ratio invest more in shoot growth to capture light for photosynthesis. In this sense, ref. [66] reported that the PAR was the most limiting factor in almond–cereal agroforestry since the shade trees may strongly affect the physiology of the undergrown crop [67]. Regarding plant height variation, no significant differences were observed between AFS and SCS. Conversely, ref. [41] asserted that this parameter was 12% significantly higher for quinoa intercropped with olive trees than sole quinoa.

### 3.2. Grain Yield and Related Yields Components

The results demonstrate that increasing salinity from 1.12 dS m^−1^ to 10.5 dS m^−1^ led to significant reductions in all parameters, except in the harvest index, across both cropping systems and experimental years. Notably, the losses were more significant in SCS compared to AFS. These outcomes corroborate the findings of [41] regarding quinoa intercropped with olive trees under supplemental irrigation at a salinity level of 6 dS.m−1. They reported that quinoa grain yield and dry matter were significantly lower in the agroforestry system (AFS) by 45% and 49%, respectively, compared to the sole cropping system (SCS). On the other hand, high salinity levels in irrigation water increased the harvest index by 11.5% when quinoa varieties were intercropped with pomegranate trees. Furthermore, the impact of salinity on all parameters was quinoa genotype dependent. Titicaca was the variety most affected by salinity, especially during the first experimental season, whereas ICBA-Q5 exhibited the lowest percentage of loss in most parameters. This finding aligns with [68], who demonstrated that increasing salinity decreased all yield and yield-related components of quinoa (cv. Titicaca).

The substantial reductions in the previous parameters due to salinity in SCS compared to AFS can be attributed to the high potential of trees in mitigating abiotic stresses such as drought and salinity. Although trees and crops may compete for scarce resources when they coexist at the same time and space, they can also assist each other through facilitation or complementarity. In addition, moderate shading and evapotranspiration from trees can improve the microclimate by reducing air temperatures and atmospheric evaporative demand [29,31,69]. Agroforestry improves soil structure and fertility by increasing organic matter and stimulating microbial activity [35,70], leading to improved nutrient cycling and greater nutrient availability for crops [38]. This can help plants cope with saline conditions by providing them with the necessary nutrients to maintain growth and productivity. Furthermore, in AFS, water and nutrient acquisition by crops may be facilitated by the trees’ hydraulic lift mechanism (HL). This process involves the redistribution of water and nutrients from deeper, wetter soil layers to the stressed upper layers, thereby enhancing the absorption of these resources by crop roots [27,42,71].

Likewise, the 11.5% increase in the harvest index under saline irrigation, observed when quinoa varieties were intercropped with pomegranate trees, implies a greater allocation of the plant’s biomass to the harvested parts. This aligns with the observed higher specific leaf area (SLA) and lower root-to-shoot ratio under saline irrigation, leading to optimal photosynthesis and seed filling under saline conditions. Additionally, the reduction in quinoa water productivity (QWP) due to salinity (10.5 dS m^−1^) was 23.9% in AFS compared to 48.6% in SCS, demonstrating greater water use efficiency in AFS. Furthermore, there were strong and positive correlations between QWP and quinoa grain yield (r = 0.84, *p* < 0.001), total dry matter (r = 0.72, *p* < 0.001), and total kernel weight (r = 0.36, *p* < 0.05).

### 3.3. Land Equivalent Ratio (LER)

The foremost advantage of agroforestry (AFS) dwells in its potential to enhance land productivity by utilizing resources more efficiently than sole cropping system (SCS). Indeed, AFS typically demonstrates a higher land equivalent ratio (LER) than monoculture crops or tree orchards. In our experiment, despite the low grain yields, LER was always more than 1, corroborating the greater efficiency of agroforestry systems rather than sole stands [72,73]. It averaged from 1.86 to 2.21, reflecting that productivity was 86 to 121% higher in AFS than in SCS. The LER of the entire system was positively influenced by the salinity level of the irrigation water (10.5 dS.m^−1^) and the specific quinoa variety associated with the pomegranate trees. It was 20% higher under saline water and reached 2.21 when associated with the ICBA-Q5 variety.

LER_Quinoa_ was significantly higher by 36.2% under saline conditions (10.5 dS.m^−1^) compared to the control (EC1 = 1.12 dS m^−1^). LER_Quinoa_ ranged from 1.08 to 1.15, indicating that quinoa component contributed between 108% and 115% to the total productivity. Likewise, the LER_Pomegranate_ varied significantly from 0.79 (P-Puno) to 1.11 (P-ICBA-Q5), indicating pomegranate’s contribution of between 79 and 111% to the global production. The partial LER_Quinoa_ consistently exceeded 1, whereas the LER_Pomegranate_ was below 1, except when pomegranate was paired with ICBA-Q5. This indicates that agroforestry was more beneficial to quinoa than to pomegranate when associated with Puno, Titicaca, and ICBA-Q4 varieties. This result supports the findings of Abidi et al. (2024) [41] when intercropping quinoa varieties with olive trees under supplemental saline irrigation (6 dS m^−1^). They found an LER averaging from 1.57 to 2.07, with overall productivity being 57% to 107% higher in the agroforestry system compared to monoculture. When intercropping quinoa with pomegranate, even under more saline conditions (10.5 dS m^−1^), LER was higher, and the overall productivity in agroforestry ranged from 86% to 121%. This comparison showed that under saline conditions, intercropping quinoa with pomegranate seems more beneficial for the whole system than intercropping quinoa with olive trees. This finding may be related to the high salt tolerance of pomegranate comparatively to olive trees.

Similar findings were reported [74,75,76,77,78] when growing other annual crops within fruit-tree-based agroforestry. This outcome resulted in a lower yield reduction due to salinity in agroforestry systems compared to sole cropping systems, highlighting the significant role of agroforestry in mitigating abiotic stresses such as salinity and drought [20,70,79,80,81,82]. In fact, saline irrigation may provide a viable solution to water scarcity when intercropping salt-resistant crops like quinoa with pomegranate trees, as both can endure moderately saline conditions. As reported by Hussin et al. (2023) [83], quinoa has an exceptional ability to regulate soil salinity thanks to its osmotic adjustment role. Additionally, agroforestry creates a preferred microclimate for the development of quinoa by reducing evapotranspiration, increasing soil structure and fertility, and enhancing nutrient and water availability [84].

Pomegranate-based agroforestry is expected to play a pivotal role under moderate saline conditions. Pomegranate is tolerant to moderate salinity. However, integrating eco-resilient crops like quinoa (*Chenopodium quinoa*, Willd.) into pomegranate-based agroforestry in saline regions represents an innovative step to enhance the salt-affected land efficiency by increasing the global LER up to 1. Our findings show that this new model of agroforestry is quinoa genotype dependent as it was profitable to pomegranate exclusively when it was associated with ICBA-Q5 (LER equal to 1.114). Indeed, the Pomegranate-ICBA-Q5 combination resulted in an increase in the average pomegranate yield by 13% and 10% compared to the pomegranate orchard under EC1 and EC2 conditions, respectively. We can suggest that under saline conditions, pomegranate trees can leverage the high potential of intercropped halophytes like quinoa to remove regulate soil salinity, thus improving pomegranate yield.

### 3.4. Quinoa Seeds Quality Profile

Quinoa exhibits exceptional nutritional properties, including high levels of minerals, protein, fats, and dietary fiber [47,53,85]. Although it thrives well in salt-afflicted areas, its nutritional components remain varying with salinity levels, genetic diversity, pedoclimatic conditions, and their interactions [86]. Furthermore, numerous studies also provide strong evidence for the potential benefits of species intercropping in enhancing crop quality [87]. In our study, intercropping quinoa with pomegranate resulted in a significant increase in the average seed contents in mineral (+9.5%), protein (+9%), and total sugar (+11.3%). This result aligns with recent findings by [41], demonstrating a 4% increase in the protein content of quinoa seeds in olive-based agroforestry compared to sole cropping systems. On the other hand, the reduction observed in the average total sugar under saline irrigation compared to the control was more pronounced in SCS (−19.2%) than in AFS (−5.6%). This is likely explained by the role of halophyte-based agroforestry in mitigating salinity pressures on photosynthates’ accumulation and nutrient uptake.

Saponins, antinutritional secondary metabolites from the glycoside family, are naturally produced by plants in response to abiotic stress [88,89,90]. These compounds endow quinoa with substantial nutraceutical potential, offering industrial and medicinal applications while also providing protection against pathogen attacks [55]. The quantity and quality of saponins in seeds are primarily determined by various environmental factors such as water availability, salinity levels, soil fertility, shading intensity, and genetic variability. In our experiment, seed saponin content varied based on the salinity level of irrigation water, cropping system, quinoa cultivar, and their interactions. The results indicated that the average seed saponin content was 18.2% lower in AFS compared to SCS. Moreover, salinity significantly impacted saponin content in AFS, unlike in SCS, across both seasons. Saponin content also varied among quinoa cultivars, ranging from 0.4% in ICBA-Q5 to 0.6% in Titicaca. These significant differences are consistent with findings by [91,92], which highlighted that saponin content is primarily a genotype-dependent trait, though it also fluctuates with abiotic stresses like salinity. Our research confirms the pivotal role of agroforestry in attenuating abiotic stresses like salinity [2,81], which can stimulate saponin synthesis [93]. AFS creates a favorable microclimate for intercropped plants, reducing competition for water and nutrients by decreasing evapotranspiration and enhancing nutrient cycling. This ensures the availability of water and nutrients for the plants. Pardon et al., 2017 [94] noted the potential of middle-aged to mature tree rows to increase soil organic carbon stocks and nutrient availability for crops in AFS. In this sense, Mouttaqi et al., 2023 [95] reported that organic amendments significantly reduced the saponin content at different salinity levels. This effect can be attributed to the amendments’ role in reducing the salinity-induced stress [96]. Additionally, Diacono and Montemurro, 2015 [97] indicated that organic amendments influence the physicochemical properties of soil due to the flocculation of minerals to organic polymers.

The cropping system contributed most to the variation in phosphorus (F = 34.8 ***), sodium (F = 7.5 **), and the K/Na ratio (F = 12.3 ***), while quinoa variety significantly affected potassium (F = 31.4 ***). Phosphorus and the K/Na ratio were significantly increased in AFS by 8.4% and 22%, respectively. Under saline irrigation (10.5 dS m^−1^), phosphorus significantly decreased by 9% and 6% in SCS over 2020 and 2021, respectively. Unlike sodium, all tailored parameters were quinoa genotype dependent [98]. Considering the ratio K/Na, Titicaca averaged a low K/Na ratio (24) while Puno, ICBA-Q4, and ICBA-Q5 did not exhibit any remarkable differences. These outcomes confirm the quinoa resistance to salinity [45,49,64]. On the other hand, in experiments where quinoa was conducted as sole crop, ref. [95] reported a high accumulation of sodium on quinoa leaves under high-salinity conditions, resulting in an increase in the K/Na ratio. Oumasst et al. (2022) [99] observed that sodium content increased tenfold under 10 dS m^−1^ of irrigation water salinity compared to 0.9 dS m^−1^ salinity level. Conversely, ref. [100] reported an increase by 60% in sodium and 65% in potassium, without any significant effect on the ratio of K/Na, under high-salinity conditions (17 dS m^−1^) compared to the control (5 dS m^−1^). Additionally, ref. [83] reported that quinoa, as a facultative halophyte, displays an effective control mechanism for xylem Na+ loading and superior K+ retention, resulting in a higher K+/Na+ ratio compared to staple crops [101]. Importantly, incorporating quinoa into pomegranate-based agroforestry systems can effectively alleviate the adverse effects of salinity on the mineral content of quinoa seeds. This approach improves soil structure and fertility, enhances water availability, and filters groundwater, thereby reducing salts accumulation in the soil and boosting seed mineral nutrient content.

### 3.5. Correlation Matrix and Principal Components Analysis

The findings of the Pearson correlation matrix underscore the nuanced influence of agricultural systems on quinoa cultivation outcomes. Sole cropping systems (SCSs) appear to foster stronger correlations among quinoa parameters, potentially indicating a more controlled environment with less competition from other plant species compared to agroforestry systems (AFSs).

Concerning the PCA, it contrasts the effects of different cropping systems and salinity levels on quinoa cultivation. For instance, quinoa plants under the lowest salinity level (C1) exhibited higher levels of grain yield, dry matter, thousand-kernel weight, and water productivity. Conversely, plants under C2 conditions showed the highest levels of seed fat content. This highlights how environmental factors like salinity can significantly influence quinoa’s agronomic and nutritional characteristics. These findings underscore the complexity of factors influencing quinoa cultivation and its yield and nutritional outcomes. Understanding these relationships can inform agricultural practices, such as optimizing cropping systems and managing environmental stressors like salinity, to enhance both yield and nutritional quality.

## 4. Materials and Methods

### 4.1. Experimental Site

**Figure 4:**
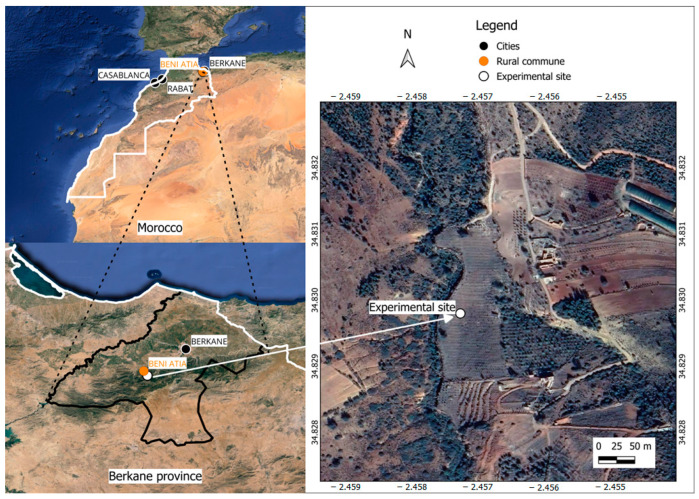
Location of the field experiment in the northeastern region of Morocco. The map was generated using QGIS (version 3.34.0).

This research was conducted over two consecutive years (2020 and 2021) in the northeastern region of Morocco, in a 15-year-old pomegranate grove. The field experiment was based in the Boughriba’s rural commune in Berkane province (34°82′98″ N 2°45′73″ W, 462 m from sea level) (Figure 4).

The soil has a silt loamy texture in the top 0–30 cm layer. In 2020 and 2021, the organic matter content averaged 2.90 and 3.04, respectively. In 2021, the soil was highly rich in phosphorus and potassium, but had lower levels of nitrogen, calcium, and magnesium than in 2020. The electrical conductivities averages were 1.22 and 0.88 in 2020 and 2021, respectively (Table 7).

**Figure 5:**
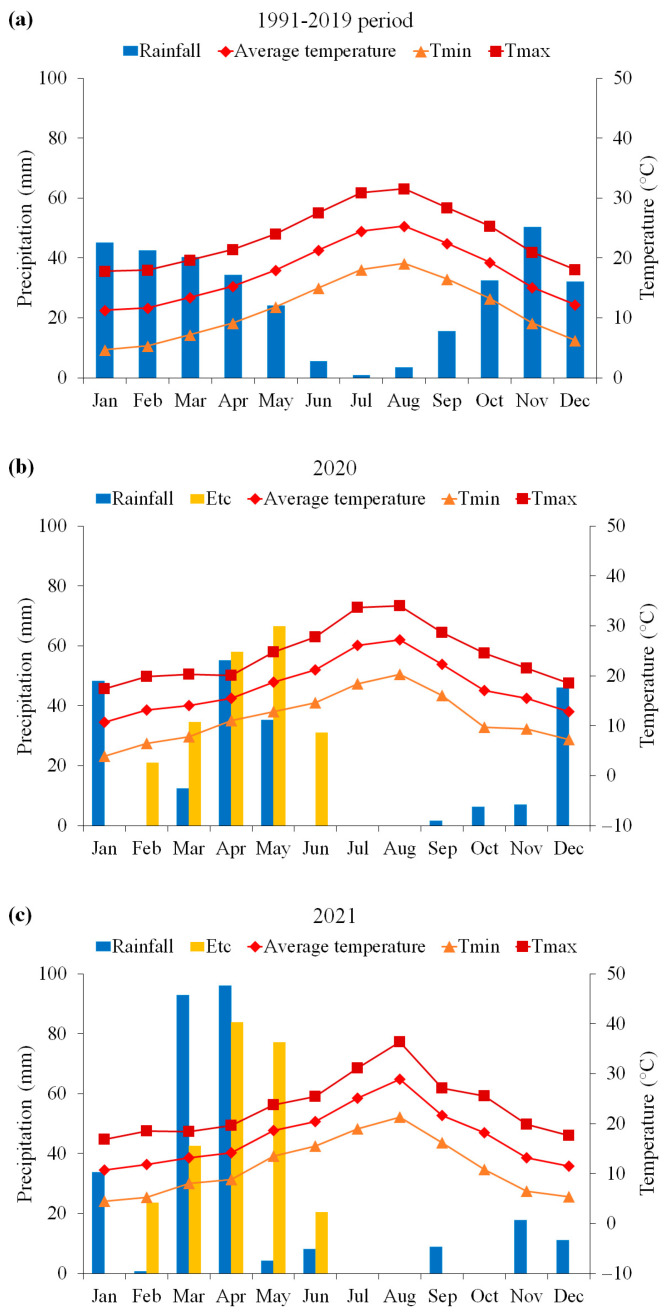
Gaussen climate diagrams illustrating the monthly precipitation patterns alongside mean, minimum, and maximum temperatures recorded during 2020 (**b**) and 2021 (**c**), in comparison to the historical 29-year mean (1991–2019) data (**a**). The yellow bars represent the quinoa water requirements (ETc) throughout the growing seasons, spanning February to June for both experimental seasons.

The climate is typically semiarid with an irregular annual rainfall averaging 325 mm over a 29-year period (CV = 28%). The average minimum and maximum temperatures were 11.32 and 23.65 °C, respectively (Figure 5a).

In 2020 and 2021, the total annual rainfall was 211.90 mm and 273.30 mm, respectively. During the quinoa growing season (from February to June), the corresponding values were 102.70 mm and 202 mm. Comparatively to the long-term average (146.86 mm), we observed a variation of −30% and +37% in cumulative rainfall during the quinoa growing season for 2020 and 2021, respectively. In 2020, the mean air temperature was 16.54 °C, while in 2021, it averaged 15.67 °C. The highest absolute temperatures occurred in June for both years, reaching 27.80 °C and 25.40 °C, respectively. The corresponding absolute minimum values were recorded in February, with 6.50 °C and 5.20 °C, respectively.

As depicted by Figure 5a, drought conditions commonly occur in May and June during the quinoa growing season. Nonetheless, in 2020, quinoa experienced a highly uneven distribution of rainfall with 0.00% in February, 65.7% in March and April, and 34.10% in May and June of total rainfall, while in 2021, quinoa received 0.3%, 93.6%, and 6% of total rainfall during the same periods. Based on our study, the three periods correspond to the main quinoa growing stages: plant establishment, panicle emergence–flowering, and grain filling–maturity. Furthermore, the crop water requirements (ETc) underscore the persistent drought conditions throughout the entire quinoa growing season in 2020 (Figure 5b) as well as during both the initial and the final stages in 2021 (Figure 5b,c).

### 4.2. Plant Material and Experimental Layout

In our experiment, the density of pomegranate trees (*Punica granatum* L.) was 278 trees ha^−1^ with a regular 6 m × 6 m plantation design. Before the experiment, the inter-rows remained uncultivated, and the pomegranate trees received occasional gravity-fed irrigation to supplement the rainfall supply. We tested four quinoa cultivars (Puno, Titicaca, ICBA-Q5, and ICBA-Q4) characterized by short growing cycles (90 to 120 days), good performance, and large-scale adaptation under semiarid conditions in Morocco [102,103,104].

**Figure 6:**
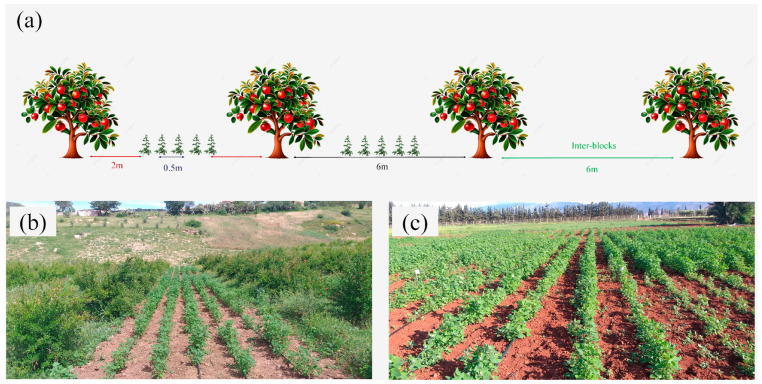
Experimental layout showing (**a**) five rows of quinoa plants spaced by 0.5 m and intercropped with pomegranate trees on the two sides of the median row of pomegranate trees in the unit plot. An empty space of 6 m was left between two adjacent blocks. A 2 m distance was left between the outer quinoa strip and the tree lines. (**b**) Quinoa–pomegranate-based agroforestry where quinoa rows were parallel to the pomegranate trees, with an east–west orientation. (**c**) Quinoa rows sown within a sole crop system (SCS), 200 m apart from the AFS grove.

During both experimental seasons (2020 and 2021), quinoa genotypes were intercropped with pomegranate trees following a consistent experimental layout. We evaluated the performance of two pomegranate agroforestry systems (AFSs) under two saline irrigation levels, with respective average electrical conductivity (EC) values of 1.12 dS m^−1^ and 10.5 dS m^−1^.The irrigation water used was issued from two available groundwater resources, Tagma’s source in Tafoghalt village was used as a control treatment, and water drilling was used as a salt treatment. We compared agroforestry systems to corresponding sole crops (SCSs) and pure pomegranate orchard (OR) used as controls. The (AFS) and (OR) were conducted under the two water regimes in the same pomegranate grove. The sole quinoa cultivars were sown in an adjacent open field plot for each irrigation water salinity level, 200 m apart to avoid any interactions between trees and sole quinoa systems. Both AFS and SCS followed a randomized complete block design with 3 replications. For AFS, quinoa varieties were sown on either side of the middle pomegranate tree row (Figure 6). In the pomegranate grove, each water regime was allocated to 15 subplots, each covering 144 m^2^ (12 m × 12 m). Among these subplots, 12 were designated for agroforestry systems while the remaining 3 subplots were assigned to pomegranate orchards. The quinoa rows were oriented east–west, parallel to the pomegranate tree rows. Similarly, for each water regime, 12 subplots of 36 m^2^ (6 m × 6 m) were dedicated to the sole crop systems (SCSs). Two inter-pomegranate rows and an empty space (12 m) were left between the two regimes in AFS and in SCS, respectively, to avoid susceptible underground interactions between quinoa varieties conducted under different regimes. The total area assigned to the experiment was 8720 m^2^.

In both AFS and SCS, quinoa varieties were meticulously hand-sown, with an inter-row and intra-plants spacing of 0.5 m and 0.20 m, respectively, using an equal sowing rate of 5 kg ha^−1^. In the AFS, a deliberate 2 m gap was maintained between pomegranate tree and quinoa plants. In 2020, quinoa was sown on 23 February and harvested between 1 and 8 July. In 2021, sowing took place on 20 February while the harvest spanned from 3 to 8 July. Following traditional practices, local organic manure (10 t ha^−1^) was applied, and weeds were manually managed. A supplemental drip irrigation was adopted, with 2 L h^−1^ integral drippers spaced 0.3 m apart. The crop water requirement was estimated based on Formula (1).

ETC = Kc × ET_0_ (1)

where kc is the quinoa crop coefficient factor, being 0.5 at plant establishment and 1 during flowering and seed filling [105]. ET_0_ is the reference evapotranspiration determined using the Penman–Monteith formula [106], based on climatic parameters collected from nearby local meteorological station.

The total volume of supplement irrigation water was estimated to 152 mm and 175 mm during the first and second cropping seasons, respectively. The corresponding total amount of water received by quinoa varieties (rainwater + irrigation) was estimated to be 254.6 and 377.4 mm, respectively. Given the 200 m average distance between the AFS and SCS plots, quinoa varieties in both systems received the same amount of rainfall. The crop coefficient (Kc) values for quinoa were consistent across both systems, whether grown solely or intercropped with pomegranate trees. We observed a slight advancement in quinoa growth within the AFS compared to the SCS, but the Kc remained unchanged, being 0.5 during plant establishment and 1.0 during flowering and seed filling. For ETC calculations, we relied on the ET_0_ value from the local meteorological station. Consequently, ETC values for all treatments were similar.

### 4.3. Field Measurements and Sampling

At the full floraison stage, 10 quinoa plants from each unit plot (5 on each side of the median pomegranate row), were entirely sampled (with root) in AFS. Similarly, in SCS, 10 rooted plants were randomly sampled from the middle of each elementary plot. The average plant height was previously determined, and root and shoot organs were separately weighted. Then, they were oven dried (70 °C, 48 h) until a constant weight of dry matter was reached. The measured parameters were root dry matter (RDM), leaf dry matter (LDM), total dry matter (TDM, leaf area (LA), specific leaf area (SLA), and root-to-shoot ratio. Specific leaf area (SLA) is calculated as the ratio of the leaf area to the leaf dry mass (SLA = leaf area/leaf dry mass). The root-to-shoot ratio is calculated as the ratio of the dry mass of the roots to the dry mass of the shoots (root-to-shoot ratio = root dry mass/shoot dry mass). These measurements were exclusively performed in 2021 due to the limited mobility during the corona virus lockdown in 2020.

At quinoa maturity, in each elementary plot, sampling was randomly performed over 3 m^2^ on either side of the median row of pomegranate trees, avoiding the borders. Afterward, quinoa plants were threshed, sorted by organ, oven dried (70 °C, 48 h), and weighed to determine the total aboveground biomass, the grain yield, and the thousand-kernel weight. The harvest index was calculated as the ratio between the grain yield and total aboveground biomass. Similarly, in the sole crop system (SCS), a sample of 2 m^2^ per elementary plot was served to determine all the monitored parameters. Moreover, upon reaching maturity, three pomegranate trees were meticulously selected from the central area of each plot unit to mitigate any border influences. Subsequently, they were handpicked to precisely evaluate the pomegranate yield per tree.

### 4.4. Quinoa Water Productivity (QWP) and Land Equivalent Ratio (LER)

Quinoa water productivity was calculated as the ratio between the obtained quinoa yield and the total amount of water supplied, encompassing both natural precipitations and supplemental irrigation applied throughout the quinoa growth cycle. To assess land use efficiency, we evaluated the land equivalent ratio (LER) as the relative land area required for sole crops and trees to achieve the same total yield as [107]. LER was calculated as the sum of relative yields in agroforestry compared to the sole crop and the orchard tree yields (Formulas (2)–(4)).

*LER*_*AFS*_ = *LER*_*Olive*_ + *LER*_*Quinoa*_ (2)

*LER*_*Olive*_ = *Olive yield*_*AFS*_/*Olive yield* (3)

*LER*_*Quinoa*_ = *Quinoa yield*_*AFS*_/*Quinoa yield*_*scs*_ (4)

The LER indicates higher (or lower) productivity for an agroforestry system (AFS) than the corresponding orchard (OR) and sole crop (SCS) when its value is above (or below) 1. When this value is equal to 1, the agroforestry system has no significant impact on land productivity [108].

### 4.5. Biochemical Analysis of Quinoa Seeds

#### 4.5.1. Grain Protein, Fat, Gross Cellulose, and Mineral Contents

The analysis of chemical and biochemical parameters was performed at the laboratory of the National Institute of Agronomic Research (INRA) in Rabat. The seed’s protein content was calculated using Formula (5), considering that most proteins contain 16% nitrogen [109].

Protein (% dry matter) = N content (% dry matter) × 6.25(5)

where 6.25 is the conversion factor used to transform nitrogen into protein. Total nitrogen content was analyzed according to the Kjeldahl procedure (AOAC, 945.18).

The fat seed content was determined using a Soxhlet extractor, while the cellulose content was performed through the application of the Weende analytical procedure (Weende, 1993). Regarding the grain mineral content analysis, we used the protocol as described by [110]. The mineral matter was determined after the calcination of the dry samples at 550 °C.

#### 4.5.2. Total Saponin Content

Saponins were isolated utilizing a slightly modified version of the protocol described in reference [111]. Briefly, 5 g of quinoa seed powder were encased in a filter paper cartridge for defatting using the Soxhlet apparatus with hexane (1:10 *w*/*v*) as a solvent. Ultrasound-assisted extraction of saponins was performed with methanol (1:10 *w*/*v*). Extraction was carried out using an ultrasonic probe at 60% amplitude for 15 min (3 cycles of 5 min). The mixture was filtered through Whatman paper N°1. The mixture was centrifuged, and the supernatant was dried under vacuum and then reconstituted in 5 mL of methanol.

The overall saponin content was determined using the modified technique described by [112]. Within this method, 0.25 mL of the saponin extract was combined with 1 mL of a reagent mixture (comprising glacial acetic acid and sulfuric acid in a 1:1 *v*/*v* ratio) and subjected to vortexing. The mixture was then incubated at 60 °C for 30 min within a thermostatically controlled water bath, followed by cooling in an ice bath. A UV–visible spectrophotometer (VWR International, Radnor, PA, USA) was utilized to measure the absorbance of the sample at 527 nm. Calibration curve preparation involved using oleanolic acid within the concentration range of 0 to 1000 μg mL^−1^. The expression of the total saponin content was in grams of oleanolic acid equivalent per 100 g of dry weight (DW).

### 4.6. Statistical Analysis

Statistical analyses were performed using the R programming language (R Core Team, Vienna, Austria, 2021) [113]. The additive model of the analysis of variance (ANOVA) was used to assess the effects of the studied factors (years, cropping system, water salinity levels, and quinoa genotypes) on the monitored parameters (agronomic and biochemical parameters). The aov package (version) of R was used for this purpose. The post hoc test was performed using the Student–Newman–Keuls test at 5% level. Pearson’s correlation matrix was performed to investigate the strength of the linear relationship between the investigated parameters, with values ranging from −1 (perfect negative correlation) to 1 (perfect positive correlation) and 0 (no linear correlation). The matrix graphical representation was carried out using the corrplot package. Additionally, principal component analysis (PCA) was served to explore the correlation between the traits and evaluate the impact of the factors on the identified correlation patterns. To perform the PCA, the “FactomineR” package was used. The visual representation of the PCA was carried out with the “Factoextra” package, and the factors were projected as supplementary qualitative variables.

## 5. Conclusions

Climate change is amplifying salinization in arid regions, threatening biodiversity as staple crops such as cereals and legumes struggle to thrive under such conditions. This trend is poised to severely affect agricultural productivity, economic yields, and food security, risking vulnerable local populations, thereby exacerbating their instability. We harness the potential of halophyte-based agroforestry to mitigate abiotic stresses, including salinity, by intercropping quinoa with pomegranate trees. We investigate how pomegranate trees’ microclimate affects quinoa’s agronomic and biochemical traits under saline irrigation. We also evaluated the system’s land use efficiency using the land equivalent ratio (LER). Our findings show an LER greater than one, indicating that the quinoa–pomegranate-based agroforestry system may be a potential agroecological solution for enhancing land use efficiency, sustaining agrobiodiversity, improving ecosystem services, and supporting local farmers in vulnerable environments to improve their livelihoods. However, further research is needed to confirm these outcomes. For instance, future studies should consider salinity’s impact on soil fertility, economic comparisons between agroforestry and monocropping, optimizing saline irrigation for quinoa’s growth cycle and targeting critical periods during the quinoa growing cycle, and utilizing organic amendments for water retention. Additionally, understanding intercropping cycles of halophytes like quinoa is crucial to avoid competition with companion trees, by avoiding overlap in their needs both spatially and temporally.

Furthermore, under an evolving climate, harnessing the halophyte-based agroforestry’s potential in mitigating salt stress in salt-afflicted lands looks promising for future climate smart agriculture and global food security. However, this requires more supportive policies and institutional frameworks to encourage farmer involvement. By integrating these strategies into political agendas, governments can foster a conducive environment for the widespread adoption of agroforestry.

## Figures and Tables

**Table 1:** Quinoa growth parameters at flowering stage as affected by the three monitored factors: cropping systems, irrigation water salinity levels, quinoa varieties, and their interactions.

		Height (cm)	TDM (g Plant^−1^)	RDM (g Plant^−1^)	LDM (g Plant^−1^)	LA (cm^2^)	SLA (cm^2^ g^−1^)	Root/Shoot
F	CS	0.2 ns	3.9 ns	5.7 *	589.5 ***	0 ns	47.6 ***	37.7 ***
	EC	41.3 ***	379.9 ***	248 ***	146 ***	3.2 ns	0.4 ns	9.4 **
	Variety	157.4 ***	256.7 ***	133.6 ***	20.9 ***	3.8 *	2.9 ns	150.3 ***
	CS × EC	0 ns	1.3 ns	0.2 ns	72.1 ***	0 ns	0.1 ns	0.8 ns
	CS × Variety	1.5 ns	1 ns	0.5 ns	20.8 ***	0 ns	1.1 ns	1.3 ns
	EC × Variety	3.9 *	121.4 ***	83.7 ***	13.4 ***	6.8 **	5.6 **	143.4 ***
	CS × EC × Variety	1.8 ns	0.8 ns	0.4 ns	22.7 ***	0 ns	3.3 *	1.3 ns
CS	AFS	102.8 ± 19.1 a	52.8 ± 28.3 a	5.1 ± 3.7 b	9.4 ± 3.9 a	5463.3 ± 2987.1 a	593.8 ± 231.5 b	0.11 ± 0.05 b
	SCS	103.5 ± 20.6 a	50.2 ± 24.9 a	5.7 ± 3.6 a	3.2 ± 0.7 b	5526.1 ± 2956.2 a	1776 ± 1014.6 a	0.13 ± 0.05 a
EC	EC1	108.4 ± 21.6 a	64.5 ± 28.6 a	7.3 ± 4.3 a	7.9 ± 5 a	6098.3 ± 3336.4 a	1132.5 ± 987.7 a	0.13 ± 0.07 a
	EC2	97.8 ± 16.4 b	38.4 ± 16.4 b	3.6 ± 1.2 b	4.8 ± 2.4 b	4891 ± 2409 a	1237.2 ± 894.9 a	0.12 ± 0.04 b
Variety	Titicaca	95.1 ± 7.6 c	45.3 ± 12.8 c	6.9 ± 4 b	7.6 ± 5.5 a	6001.6 ± 3288.7 a	1221.1 ± 987.6 ab	0.17 ± 0.07 a
	Puno	131.7 ± 12 a	80.3 ± 32.4 a	8.5 ± 3.7 a	7 ± 4.5 a	5904.9 ± 3446.4 a	1193 ± 981 ab	0.12 ± 0.01 c
	ICBA-Q4	102.7 ± 6.6 b	51.5 ± 6.7 b	3.2 ± 1 c	5.7 ± 2.5 b	6491.4 ± 1998 a	1517.2 ± 1073.4 a	0.07 ± 0.02 d
	ICBA-Q5	83.1 ± 7.1 d	28.9 ± 14.6 d	3.1 ± 1 c	5.1 ± 3.1 b	3580.7 ± 1886.9 b	808.2 ± 469 b	0.14 ± 0.04 b

Values are means ± standard deviation (n = 12). In each factor, for each parameter, means followed by the same lowercase letters are not significantly different. * denotes *p* < 0.05, ** denotes *p* < 0.01, *** denotes *p* < 0.001; ns = not significant. CS, AFS, and SCS are cropping system, agroforestry, and sole cropping systems, respectively. EC1 and EC2 are fresh water (1.12 dS.m^−1^) and saline water (10.5 dS.m^−1^) for irrigation, respectively. The statistical analysis was performed using R programming language. TDM: total dry matter, RDM: root dry matter, LDM: leaf dry matter, LA: leaf area; SLA: specific leaf area.

**Table 2:** Grain yield and yield-related components as affected by the monitored factors and their interactions.

		Dry Biomass (t ha^−1^)	Grain Yield (t ha^−1^)	Harvest Index	Thousand-Kernel Weight (g)	Quinoa Water Productivity (kg m^−3^)
F	Year	140.2 ***	51.7 ***	0 ns	32.7 ***	61.3 ***
	CS	0.7 ns	3.4 ns	5 *	38.8 ***	3.5 ns
	EC	944.3 ***	266.7 ***	10.7 **	82.9 ***	265.8 ***
	Variety	12.6 ***	5.1 **	14.4 ***	121.1 ***	5.1 **
	Year × EC	10.6 **	2.9 ns	0 ns	0.2 ns	2.2 ns
	CS × EC	48.9 ***	32.5 ***	8 **	5.6 *	32 ***
	CS × Variety	1.5 ns	1.8 ns	3.5 *	0.7 ns	1.8 ns
	EC × Variety	35.2 ***	7.5 ***	11.7 ***	4.9 **	7.6 ***
	CS × EC × Variety	11.2 ***	4.8 **	4.7 **	0.3 ns	4.9 **
Year	2020	4.7 ± 1.39 b	2 ± 0.95 b	0.45 ± 0.19 a	2.4 ± 0.57 a	0.79 ± 0.37 a
	2021	5.6 ± 1.58 a	2.4 ± 1.16 a	0.45 ± 0.19 a	2.3 ± 0.51 b	0.64 ± 0.31 b
CS	AFS	5.2 ± 1.31 a	2.3 ± 0.98 a	0.46 ± 0.19 a	2.5 ± 0.57 a	0.73 ± 0.32 a
	SCS	5.1 ± 1.76 a	2.2 ± 1.17 a	0.44 ± 0.19 b	2.3 ± 0.51 b	0.7 ± 0.38 a
EC	EC1	6.4 ± 1.09 a	2.7 ± 1.16 a	0.44 ± 0.19 b	2.5 ± 0.56 a	0.86 ± 0.38 a
	EC2	3.9 ± 0.76 b	1.7 ± 0.74 b	0.46 ± 0.19 a	2.2 ± 0.5 b	0.56 ± 0.24 b
Varieties	Titicaca	5.3 ± 2.02 a	2.5 ± 0.81 b	0.5 ± 0.07 b	2.9 ± 0.35 a	0.8 ± 0.26 b
	Puno	4.9 ± 1.47 b	2.8 ± 0.97 a	0.6 ± 0.05 a	2.5 ± 0.38 c	0.9 ± 0.32 a
	ICBA-Q4	4.7 ± 1.22 c	2.5 ± 0.64 b	0.5 ± 0.06 a	2 ± 0.19 d	0.8 ± 0.21 b
	ICBA-Q5	4.7 ± 0.93 c	2.6 ± 0.54 b	0.6 ± 0.05 a	2.8 ± 0.26 b	0.9 ± 0.17 b

Values are means ± standard deviation (n = 24). In each factor, for each parameter, means followed by the same letters are not significantly different. * denotes *p* < 0.05, ** denotes *p* < 0.01, *** denotes *p* < 0.001; ns = not significant. CS, AFS, and SCS are cropping system, agroforestry, and sole cropping systems, respectively. EC1 and EC2 are electrical conductivities of fresh water (1.12 dS.m^−1^) and saline water (10.5 dS.m^−1^) for irrigation, respectively. The statistical analysis was performed using R programming language.

**Table 3:** Variation (%) in agronomic parameters due to the irrigation water salinity EC2 (10.5 dS m^−1^) compared to the control EC1 (1.12 dS m^−1^) in each cropping system across both experimental seasons.

Year	CS	DM (%)	Grain Yield (%)	HI (%)	TKW (%)	QWP (%)
2020	AFS	−30.1	−28	11.5	−10.3	−23.9
	SCS	−45.6	−46.7	0	−14.8	−48.6
	Mean 2020	−37.8	−34.5	5.7	−10.7	−36.4
2021	AFS	−31.3	−24.2	11.5	−7.7	−23.9
	SCS	−47.1	−45.9	0	−16	−46.4
	Mean 2021	−40.3	−34.3	5.7	−11.5	−33.3
Overall mean		−39.3	−34.4	5.7	−11.1	−34.9

CS, AFS, and SCS are cropping system, agroforestry, and sole cropping systems, respectively.

**Table 4:** Partial and combined land equivalent ratios as affected by irrigation water salinity and quinoa variety over two experimental seasons.

		LER_Quinoa_	LER_Pomegranate_	LER
F	Year	0.1 ns	4.9 *	0.6 ns
	EC	108.6 ***	1.9 ns	85.3 ***
	Variety	1.1 ns	198.3 ***	10 ***
	Year × EC	0.2 ns	4.9 *	0 ns
	Year × Variety	0 ns	0.4 ns	0 ns
	EC × Variety	6.9 ***	0.7 ns	6.3 ***
	Year × EC × Variety	0 ns	0.4 ns	0 ns
Year	2020	1.126 ± 0.232 a	0.901 ± 0.14 a	2.027 ± 0.275 a
	2021	1.115 ± 0.237 a	0.887 ± 0.144 b	2.003 ± 0.268 a
EC	EC1	0.949 ± 0.179 b	0.902 ± 0.144 a	1.851 ± 0.258 b
	EC2	1.293 ± 0.127 a	0.886 ± 0.14 a	2.179 ± 0.16 a
Associations	P-Titicaca	1.154 ± 0.194 a	0.861 ± 0.073 b	2.015 ± 0.241 b
	P-Puno	1.077 ± 0.322 a	0.786 ± 0.049 d	1.863 ± 0.326 b
	P-ICBA-Q4	1.161 ± 0.228 a	0.815 ± 0.044 c	1.976 ± 0.232 b
	P-ICBA-Q5	1.091 ± 0.176 a	1.114 ± 0.036 a	2.205 ± 0.159 a

Values are means ± standard deviation (n = 12). For each experimental year and salinity level and system, means followed by the same lowercase letters are not significantly different. * denotes *p* < 0.05, *** denotes *p* < 0.001; ns = not significant. AFS and SCS are agroforestry and sole cropping systems, respectively. The statistical analysis was performed using R programming language.

**Table 5:** Variation in quinoa seeds’ nutrients and saponin content according to experimental season, cropping system, irrigation water salinity, quinoa genotypes, and their interactions.

		Mineral (% DM)	Protein (% DM)	Fat (% DM)	CB (% DM)	Total Sugar (mg 10g^−1^ DM^−1^)	Saponin (g 100g^−1^ DM^−1^)
F	Year	12 ***	0.4 ns	3.2 ns	0.2 ns	0.3 ns	0.4 ns
	CS	178.2 ***	84.5 ***	1.4 ns	0.3 ns	39.9 ***	9.6 **
	EC	14.5 ***	1.4 ns	0.4 ns	0.2 ns	63.4 ***	1.9 ns
	Variety	5.9 **	4.8 **	15.9 ***	21.8 ***	17.2 ***	4.3 **
	Year × CS	77.5 ***	14.8 ***	0 ns	0 ns	0 ns	0.8 ns
	Year × EC	13.3 ***	0.5 ns	0 ns	0 ns	0 ns	0.7 ns
	CS × EC	3.4 ns	0.4 ns	2.3 ns	0.2 ns	19.4 ***	12.2 ***
	EC × Variety	6 **	2 ns	10.7 ***	18 ***	1.6 ns	1.2 ns
Year	2020	89.7 ± 4.08 a	16.5 ± 1.09 a	5.5 ± 0.4 a	4.8 ± 1.02 a	243.5 ± 33.75 a	0.51 ± 0.16 a
	2021	87.6 ± 7.57 b	16.4 ± 1.27 a	5.4 ± 0.39 a	4.7 ± 1.01 a	241.1 ± 33.41 a	0.49 ± 0.15 a
CS	AFS	92.6 ± 4.5 a	17.1 ± 0.99 a	5.5 ± 0.4 a	4.8 ± 1.02 a	255.3 ± 25.49 a	0.46 ± 0.15 b
	SCS	84.6 ± 4.82 b	15.7 ± 0.9 b	5.5 ± 0.39 a	4.7 ± 1 a	229.3 ± 35.53 b	0.54 ± 0.15 a
EC	EC1	89.8 ± 5.12 a	16.5 ± 1.12 a	5.5 ± 0.43 a	4.8 ± 0.96 a	258.7 ± 27.75 a	0.52 ± 0.14 a
	EC2	87.5 ± 6.87 b	16.3 ± 1.24 a	5.5 ± 0.36 a	4.8 ± 1.06 a	225.9 ± 30.63 b	0.48 ± 0.17 a
Varieties	Titicaca	89.3 ± 5.65 a	16 ± 1.29 b	5.4 ± 0.29 b	4.3 ± 1.12 c	216.9 ± 24.83 b	0.6 ± 0.13 a
	Puno	89.3 ± 5.62 a	16.8 ± 1.34 a	5.6 ± 0.25 b	4.2 ± 0.36 c	248.5 ± 30.63 a	0.5 ± 0.2 ab
	ICBA-Q4	86.4 ± 7.34 b	16.6 ± 1.02 a	5.8 ± 0.29 a	5.1 ± 1.02 b	254.2 ± 25.93 a	0.5 ± 0.11 b
	ICBA-Q5	89.4 ± 5.6 a	16.3 ± 0.94 ab	5.2 ± 0.48 c	5.6 ± 0.55 a	249.6 ± 38.35 a	0.4 ± 0.14 b

Values are means ± standard deviation (n = 24). For each experimental year and cropping system, means followed by the same lowercase letters are not significantly different. ** denotes *p* < 0.01, *** denotes *p* < 0.001; ns = not significant. AFS and SCS are agroforestry and sole cropping systems, respectively. The statistical analysis was performed using R programming language.

**Table 6:** Mineral composition of quinoa seeds at harvest.

		P	K	Na	K/Na Ratio
F	Year	7.6 **	0.7 ns	1.6 ns	2.9 ns
	CS	34.8 ***	3.8 ns	7.5 **	12.3 ***
	EC	0.9 ns	5.4 *	0.1 ns	1 ns
	Variety	8.4 ***	31.4 ***	1.8 ns	6.2 ***
	CS × EC	18.5 ***	0.6 ns	0.5 ns	1.3 ns
	CS × Variety	4.3 **	1.9 ns	0 ns	0.4 ns
	EC × Variety	7.8 ***	1.6 ns	3.8 *	4.8 **
	CS × EC × Variety	7.6 ***	1.8 ns	0.1 ns	0.2 ns
Year	2020	0.067 ± 0.007 a	1.4 ± 0.18 a	0.1 ± 0.01 a	31.7 ± 9.74 a
	2021	0.065 ± 0.006 b	1.4 ± 0.17 a	0.1 ± 0.01 a	28.8 ± 8.61 a
CS	AFS	0.069 ± 0.008 a	1.4 ± 0.2 a	0.1 ± 0.01 b	33.2 ± 9.87 a
	SCS	0.063 ± 0.004 b	1.4 ± 0.14 a	0.1 ± 0.02 a	27.2 ± 7.6 b
EC	EC1	0.066 ± 0.004 a	1.4 ± 0.13 a	0.1 ± 0.01 a	29.4 ± 6.52 a
	EC2	0.065 ± 0.009 a	1.4 ± 0.2 b	0.1 ± 0.02 a	31.1 ± 11.37 a
Varieties	Titicaca	0.1 ± 0.01 a	1.3 ± 0.14 d	0.1 ± 0.01 a	24 ± 5.51 b
	Puno	0.1 ± 0.01 a	1.5 ± 0.17 b	0.1 ± 0.02 a	32.3 ± 12.44 a
	ICBA-Q4	0.1 ± 0.01 b	1.6 ± 0.07 a	0.1 ± 0.01 a	33.4 ± 8.34 a
	ICBA-Q5	0.1 ± 0.01 a	1.3 ± 0.09 c	0.1 ± 0.01 a	31 ± 6.57 a

Values are means ± standard deviation (n = 24). For each experimental year and cropping system, means followed by the same lowercase letters are not significantly different. * denotes *p* < 0.05, ** denotes *p* < 0.01, *** denotes *p* < 0.001; ns = not significant. AFS and SCS are agroforestry and sole cropping systems, respectively. The statistical analysis was performed using R programming language.

**Table 7:** Initial soil physical and chemical properties in the experimental site in 2020 and 2021 (0–30 cm).

	2020		2021	
	SCS	AFS	SCS	AFS
Texture	Silt loam	Silt loam	Silt loam	Silt loam
ECe (mS cm^−1^, 25 °C)	1.35	1.08	0.96	0.80
OM (%)	2.78	3.01	2.75	3.32
pH	7.38	7.12	7.35	7.13
P_2_O_5_ (ppm)	3.75	4.35	3.98	5.12
K2O (ppm)	228	265	239	2.85
NO_3_ (ppm)	2.45	3.12	2.65	3.32
Ca (mg 100 g^−1^)	658	725	665	687
Mg (mg 100 g^−1^)	129	135	131	142
Actif limestone (%)	8.28	8.22	8.25	8.18

AFS: agroforestry system; SCS: sole crop system. Soil analysis was performed at the laboratory of the National Institute for Agricultural Research in Rabat, Morocco.

## Data Availability

The data are contained within the article.

## Supplementary Materials

The following supporting information can be downloaded at: https://www.mdpi.com/article/10.3390/plants13182543/s1, Figure S1: Seeds contents of mineral, protein, fat, gross cellulose, total sugar, and saponin as affected by irrigation water salinity levels in each cropping system over each experimental season.; Table S1: Effect of irrigation water salinity on Grain yield and yield-related components of quinoa genotypes depending on cropping sys-tems over the experimental seasons; Table S2. Effect of irrigation water salinity on Phophorus, Potassium, Sodium and K/Na ratio according to quinoa genotypes, cropping systems and experimental seasons.
