# Supplementary material for: Pomegranate–Quinoa-Based Agroforestry System: An Innovative Strategy to Alleviate Salinity Effects and Enhance Land Use Efficiency in Salt-Affected Semiarid Regions
Source: Plants (Basel). 2024 Sep 10;13(18):2543. doi: 10.3390/plants13182543 (PMC11435191; doi:10.3390/plants13182543)
Supplement: Supplementary file 1 [file plants-13-02543-s001.zip › plants-3136899-supplementary.pdf]

Supplementary Table S1. Effect of irrigation water salinity on Grain yield and yield-related components of quinoa genotypes depending on cropping systems over the experimental seasons

			Dry Biomass (t ha^{-1})		Grain yield (t ha^{-1})		Harvest index		Thousand kernel weight (g)		Quinoa Water Productivity (kg m^{-3})	
			EC1	EC2	EC1	EC2	EC1	EC2	EC1	EC2	EC1	EC2
2020	AFS	Titicaca	6.1 ± 1.36 a	3.3 ± 2.53 b	2.9 ± 0.76 a	1.8 ± 1.22 b	0.48 ± 0.23 b	0.56 ± 0.2 a	3.4 ± 1.24 a	3.0 ± 1.4 a	1.15 ± 0.3 a	0.72 ± 0.48 b
		Puno	5.0 ± 1.66 a	4.0 ± 2.07 b	2.9 ± 0.89 a	2.2 ± 1.2 b	0.58 ± 0.22 a	0.54 ± 0.24 a	3.0 ± 1.06 a	2.5 ± 1.23 a	1.13 ± 0.35 a	0.84 ± 0.47 b
		ICBA-Q4	5.2 ± 1.49 a	3.6 ± 2.14 b	2.6 ± 0.92 a	2.2 ± 1.09 a	0.5 ± 0.26 b	0.62 ± 0.21 a	2.2 ± 0.85 a	2.0 ± 0.92 b	1.02 ± 0.36 a	0.87 ± 0.43 a
		ICBA-Q5	5.0 ± 1.55 a	3.7 ± 2.06 b	2.7 ± 0.94 a	2.3 ± 1.11 a	0.54 ± 0.25 a	0.60 ± 0.22 a	3.1 ± 1.2 a	2.9 ± 1.27 a	1.05 ± 0.37 a	0.89 ± 0.44 a
		**Means**	**5.3 ± 0.53 A**	**3.7 ± 0.36 B**	**2.8 ± 0.29 A**	**2.1 ± 0.22 B**	**0.52 ± 0.05 B**	**0.58 ± 0.05 A**	**2.9 ± 0.46 A**	**2.6 ± 0.44 B**	**1.09 ± 0.11 A**	**0.83 ± 0.09 B**
	SCS	Titicaca	6.8 ± 1.26 a	3.0 ± 2.81 b	2.8 ± 0.66 a	1.6 ± 1.18 a	0.40 ± 0.22 a	0.53 ± 0.17 a	3.2 ± 1.03 a	2.5 ± 1.31 b	1.08 ± 0.26 a	0.61 ± 0.46 a
		Puno	6.1 ± 1.17 a	2.8 ± 2.54 b	3.7 ± 0.65 a	1.6 ± 1.54 b	0.61 ± 0.23 a	0.56 ± 0.25 a	2.8 ± 0.91 a	2.2 ± 1.17 b	1.46 ± 0.26 a	0.62 ± 0.61 b
		ICBA-Q4	5.2 ± 1.28 a	3.1 ± 2.18 b	2.7 ± 0.67 a	1.6 ± 1.13 b	0.51 ± 0.22 a	0.52 ± 0.21 a	2.1 ± 0.8 a	1.9 ± 0.86 a	1.05 ± 0.26 a	0.63 ± 0.44 b
		ICBA-Q5	4.6 ± 1.44 a	3.5 ± 1.93 b	2.8 ± 0.76 a	1.8 ± 1.15 b	0.6 ± 0.22 a	0.53 ± 0.25 a	2.9 ± 1.12 a	2.7 ± 1.19 a	1.09 ± 0.3 a	0.72 ± 0.45 b
		**Means**	**5.7 ± 0.95 A**	**3.1 ± 0.39 B**	**3 ± 0.61 A**	**1.6 ± 0.19 B**	**0.53 ± 0.1 A**	**0.53 ± 0.04 A**	**2.7 ± 0.42 A**	**2.3 ± 0.36 B**	**1.17 ± 0.24 A**	**0.65 ± 0.07 B**
	**Means of 2020**		**5.5 ± 0.78 A**	**3.4 ± 0.47 B**	**2.9 ± 0.48 A**	**1.9 ± 0.31 B**	**0.53 ± 0.08 B**	**0.56 ± 0.05 A**	**2.8 ± 0.44 A**	**2.5 ± 0.42 B**	**1.1 ± 0.19 A**	**0.7 ± 0.12 B**
2021	AFS	Titicaca	7.4 ± 1.63 a	3.9 ± 3.07 b	3.5 ± 0.91 a	2.2 ± 1.46 b	0.47 ± 0.23 b	0.56 ± 0.2 a	3.1 ± 1.13 a	2.7 ± 1.28 a	0.93 ± 0.24 a	0.58 ± 0.39 b
		Puno	6.0 ± 2.00 a	4.8 ± 2.49 b	3.5 ± 1.07 a	2.6 ± 1.43 b	0.58 ± 0.22 a	0.54 ± 0.24 a	2.7 ± 0.96 a	2.3 ± 1.12 a	0.92 ± 0.28 a	0.68 ± 0.38 b
		ICBA-Q4	6.2 ± 1.78 a	4.3 ± 2.57 b	3.1 ± 1.1 a	2.6 ± 1.31 a	0.5 ± 0.26 b	0.62 ± 0.21 a	2.0 ± 0.77 a	1.9 ± 0.84 b	0.83 ± 0.29 a	0.70 ± 0.35 a
		ICBA-Q5	6.0 ± 1.84 a	4.4 ± 2.47 b	3.2 ± 1.12 a	2.7 ± 1.33 a	0.54 ± 0.25 a	0.61 ± 0.22 a	2.8 ± 1.2 a	2.9 ± 1.16 a	0.85 ± 0.3 a	0.72 ± 0.35 a
		**Means**	**6.4 ± 0.68 A**	**4.4 ± 0.43 B**	**3.3 ± 0.34 A**	**2.5 ± 0.27 B**	**0.52 ± 0.05 B**	**0.58 ± 0.05 A**	**2.6 ± 0.41 A**	**2.4 ± 0.46 B**	**0.88 ± 0.09 A**	**0.67 ± 0.07 B**
	SCS	Titicaca	8.1 ± 1.52 a	3.6 ± 3.37 b	3.3 ± 0.79 a	1.9 ± 1.41 a	0.40 ± 0.22 a	0.53 ± 0.17 a	3.0 ± 1.02 a	2.5 ± 1.22 b	0.87 ± 0.21 a	0.50 ± 0.37 a
		Puno	7.4 ± 1.40 a	3.4 ± 3.05 b	4.5 ± 0.78 a	1.9 ± 1.85 b	0.61 ± 0.23 a	0.56 ± 0.25 a	2.5 ± 0.85 a	2.0 ± 1.04 a	1.18 ± 0.21 a	0.50 ± 0.49 b
		ICBA-Q4	6.3 ± 1.53 a	3.7 ± 2.64 b	3.4 ± 0.81 a	1.9 ± 1.41 b	0.53 ± 0.22 a	0.52 ± 0.22 a	2.0 ± 0.67 a	1.6 ± 0.81 b	0.9 ± 0.21 a	0.51 ± 0.37 b
		ICBA-Q5	6.0 ± 1.72 a	4.2 ± 2.50 b	3.5 ± 0.91 a	2.2 ± 1.44 b	0.57 ± 0.22 a	0.53 ± 0.24 a	2.7 ± 0.94 a	2.3 ± 1.13 b	0.92 ± 0.24 a	0.58 ± 0.38 b
		**Means**	**7.0 ± 0.99 A**	**3.7 ± 0.47 B**	**3.7 ± 0.71 A**	**2.0 ± 0.22 B**	**0.53 ± 0.10 A**	**0.53 ± 0.04 A**	**2.5 ± 0.39 A**	**2.1 ± 0.39 B**	**0.97 ± 0.19 A**	**0.52 ± 0.06 B**
	**Means of 2021**		**6.7 ± 0.88 A**	**4.0 ± 0.55 B**	**3.5 ± 0.57 A**	**2.3 ± 0.38 B**	**0.53 ± 0.07 B**	**0.56 ± 0.05 A**	**2.6 ± 0.40 A**	**2.3 ± 0.45 B**	**0.90 ± 0.15 A**	**0.60 ± 0.10 B**
**Overall means**			**6.1 ± 1.01 A**	**3.7 ± 0.61 B**	**3.2 ± 0.61 A**	**2.1 ± 0.39 B**	**0.53 ± 0.08 B**	**0.56 ± 0.05 A**	**2.7 ± 0.43 A**	**2.4 ± 0.45 B**	**1.03 ± 0.20 A**	**0.67 ± 0.13 B**

Values are means ± standard deviation (n = 3). For each variety (for each parameter), means followed by the same lowercase letters are not significantly different. For each cropping system in each year (for each parameter), means followed by the same uppercase letters are not significantly different. . For each experimental year (for each parameter), means followed by the same uppercase letters are not significantly different. . For the overall means (for each parameter), means followed by the same uppercase letters are not significantly different. CS, AFS and SCS are cropping system, agroforestry and sole cropping systems, respectively. EC1 and EC2 are electrical conductivities of fresh water (1.12 dS.m−1) and saline water (10.5 dS.m−1) for irrigation, respectively. The statistical analysis was performed using R programming language.

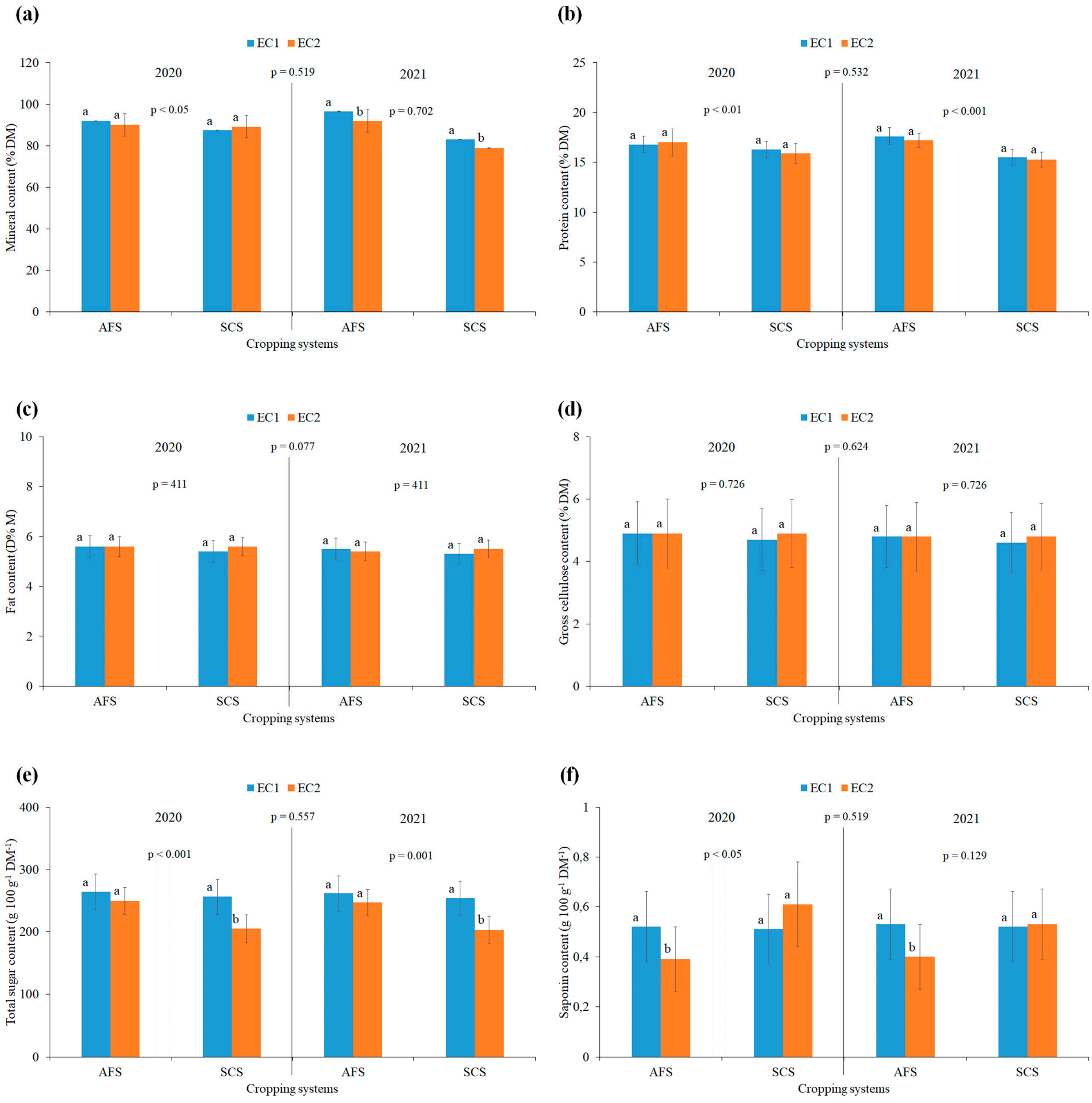

Supplementary Figure S1. Seeds contents of mineral, protein, fat, gross cellulose, total sugar, and saponin as affected by irrigation water salinity levels in each cropping system over each experimental season. Vertical bars represent standard deviation (n = 12). AFS and SCS are agroforestry and sole cropping systems, respectively.

Supplementary Table S2. Effect of irrigation water salinity on Phophorus, Potassium, Sodium and K/Na ratio according to quinoa genotypes, cropping systems and experimental seasons.

			P		K		Na		K/Na ratio	
			EC1	EC2	EC1	EC2	EC1	EC2	EC1	EC2
2020	AFS	Titicaca	0.071 ± 0.025 a	0.061 ± 0.03 b	1.4 ± 0.49 a	1.2 ± 0.56 a	0.05 ± 0.02 a	0.05 ± 0.02 a	28.3 ± 11.46 a	26.3 ± 12 a
		Puno	0.068 ± 0.033 a	0.079 ± 0.028 a	1.5 ± 0.67 a	1.6 ± 0.62 a	0.05 ± 0.02 a	0.05 ± 0.02 a	33.4 ± 19.24 a	42.9 ± 13.97 a
		ICBA-Q4	0.064 ± 0.029 a	0.07 ± 0.027 a	1.6 ± 0.68 a	1.6 ± 0.68 a	0.04 ± 0.02 a	0.05 ± 0.02 a	43.9 ± 15.14 a	34.9 ± 18.2 a
		ICBA-Q5	0.07 ± 0.031 a	0.075 ± 0.029 a	1.4 ± 0.56 a	1.3 ± 0.58 a	0.05 ± 0.01 a	0.03 ± 0.02 a	30.3 ± 17.01 a	40.7 ± 12.6 a
		**Means**	**0.069 ± 0.004 A**	**0.071 ± 0.01 A**	**1.5 ± 0.14 A**	**1.4 ± 0.27 A**	**0.04 ± 0.01 A**	**0.04 ± 0.02 A**	**34 ± 7.43 A**	**36.2 ± 12.8 A**
	SCS	Titicaca	0.07 ± 0.026 a	0.063 ± 0.029 b	1.3 ± 0.52 a	1.3 ± 0.55 a	0.06 ± 0.02 a	0.05 ± 0.03 a	23.1 ± 9.81 a	23.5 ± 9.8 a
		Puno	0.067 ± 0.025 a	0.061 ± 0.028 b	1.5 ± 0.58 a	1.4 ± 0.61 a	0.05 ± 0.03 a	0.06 ± 0.02 a	27.3 ± 14.89 a	31.8 ± 11.41 a
		ICBA-Q4	0.063 ± 0.024 a	0.058 ± 0.026 b	1.6 ± 0.63 a	1.5 ± 0.66 b	0.04 ± 0.03 a	0.06 ± 0.02 a	35.8 ± 11.47 a	26.7 ± 14.86 a
		ICBA-Q5	0.069 ± 0.026 a	0.063 ± 0.029 a	1.4 ± 0.54 a	1.3 ± 0.57 a	0.06 ± 0.02 a	0.04 ± 0.02 a	24.8 ± 13.65 a	32.6 ± 10.29 a
		**Means**	**0.067 ± 0.004 A**	**0.061 ± 0.003 B**	**1.4 ± 0.14 A**	**1.4 ± 0.13 A**	**0.05 ± 0.01 A**	**0.05 ± 0.02 A**	**27.8 ± 6.07 A**	**28.7 ± 9.72 A**
	**Means of 2020**		**0.068 ± 0.004 A**	**0.066 ± 0.009 A**	**1.5 ± 0.14 A**	**1.4 ± 0.21 A**	**0.1 ± 0.01 A**	**0.1 ± 0.02 A**	**30.9 ± 7.36 A**	**32.4 ± 11.76 A**
2021	AFS	Titicaca	0.071 ± 0.024 a	0.059 ± 0.029 b	1.3 ± 0.48 a	1.1 ± 0.56 a	0.06 ± 0.02 a	0.05 ± 0.02 a	23 ± 10.7 a	24.5 ± 9.57 a
		Puno	0.064 ± 0.032 a	0.076 ± 0.027 a	1.5 ± 0.66 a	1.6 ± 0.61 a	0.05 ± 0.02 a	0.05 ± 0.02 a	28.4 ± 17.96 a	40 ± 11.78 a
		ICBA-Q4	0.061 ± 0.028 a	0.067 ± 0.025 a	1.6 ± 0.67 a	1.6 ± 0.67 a	0.05 ± 0.02 a	0.05 ± 0.02 a	35.2 ± 14.13 a	32.6 ± 14.57 a
		ICBA-Q5	0.066 ± 0.03 a	0.072 ± 0.027 a	1.4 ± 0.55 a	1.3 ± 0.57 a	0.05 ± 0.01 a	0.04 ± 0.02 b	28.6 ± 15.88 a	38 ± 11.85 a
		**Means**	**0.066 ± 0.005 A**	**0.069 ± 0.01 A**	**1.5 ± 0.14 A**	**1.4 ± 0.26 A**	**0.05 ± 0.01 A**	**0.05 ± 0.02 A**	**28.8 ± 4.71 A**	**33.8 ± 11.95 A**
	SCS	Titicaca	0.065 ± 0.026 a	0.063 ± 0.027 a	1.3 ± 0.51 a	1.2 ± 0.55 a	0.06 ± 0.02 a	0.06 ± 0.03 a	22.5 ± 8.74 a	21 ± 9.51 a
		Puno	0.063 ± 0.025 a	0.061 ± 0.026 a	1.4 ± 0.57 a	1.4 ± 0.6 a	0.06 ± 0.03 a	0.06 ± 0.02 a	26.5 ± 13.26 a	28.4 ± 11.08 a
		ICBA-Q4	0.061 ± 0.023 a	0.056 ± 0.025 a	1.6 ± 0.62 a	1.5 ± 0.66 b	0.05 ± 0.03 a	0.07 ± 0.02 a	34.8 ± 10.22 a	23.8 ± 14.42 a
		ICBA-Q5	0.067 ± 0.026 a	0.062 ± 0.028 a	1.4 ± 0.53 a	1.3 ± 0.56 a	0.06 ± 0.02 a	0.04 ± 0.02 a	24.1 ± 12.16 a	29.1 ± 9.98 a
		**Means**	**0.064 ± 0.003 A**	**0.06 ± 0.004 B**	**1.4 ± 0.14 A**	**1.3 ± 0.13 A**	**0.05 ± 0.01 A**	**0.06 ± 0.02 A**	**26.9 ± 5.89 A**	**25.5 ± 8.66 A**
	**Means of 2021**		**0.065 ± 0.004 A**	**0.065 ± 0.008 A**	**1.4 ± 0.14 A**	**1.4 ± 0.2 A**	**0.1 ± 0.01 A**	**0.1 ± 0.02 A**	**27.9 ± 5.3 A**	**29.7 ± 11.04 A**
**Overall means**			**0.066 ± 0.004 A**	**0.065 ± 0.009 A**	**1.4 ± 0.13 A**	**1.4 ± 0.2 B**	**0.1 ± 0.01 A**	**0.1 ± 0.02 A**	**29.4 ± 6.52 A**	**31.1 ± 11.37 A**

Values are means ± standard deviation (n = 3). For each experimental year and cropping system, means followed by the same lowercase letters are not significantly different. *, **, *** indicate the significance level at $p < 0.05$, 0.01, and 0.001, respectively. AFS and SCS are agroforestry and sole cropping systems, respectively. The statistical analysis was performed using R programming language.
